# SARS-CoV‑2-Zufallsentdeckungen bei Hamburger Todesfällen: ein epidemiologisches Monitoring während des dynamischen Infektionsgeschehens im Frühjahr 2020

**DOI:** 10.1007/s00194-021-00481-w

**Published:** 2021-04-20

**Authors:** Anke Klein, Felicia Langenwalder, Fabian Heinrich, Kira Meißner, Ann Sophie Schröder, Klaus Püschel, Benjamin Ondruschka, Marc Lütgehetmann, Axel Heinemann

**Affiliations:** 1 Institut für Rechtsmedizin, Universitätsklinikum Hamburg-Eppendorf, Butenfeld 34, 22529 Hamburg, Deutschland; 2 Institut für Medizinische Mikrobiologie, Virologie und Hygiene, Universitätsklinikum Hamburg-Eppendorf, Martinistr. 52, 20246 Hamburg, Deutschland

**Keywords:** Coronavirus, COVID-19, Epidemiologie, Mortalität, Dunkelziffer, Corona virus, COVID-19, Epidemiology, Mortality, Estimated number of unreported cases

## Abstract

**Hintergrund:**

Im Rahmen der COVID-19-Pandemie sind Inzidenz und Mortalität entscheidende Determinanten, um Ausbreitungsdynamik und Gefahrenpotenzial zu beurteilen. Untersucht wird, ob ein systematisches mikrobiologisches Monitoring von Todesfällen eine relevante Untererfassung der Mortalität aufzeigen kann, und ob sich ggf. eine Sterbekohorte zuvor nicht erfasster Fälle von einer Hellfeldkohorte unterscheidet (Soziografie, Todesursache).

**Methode:**

Es erfolgte eine systematische Reverse-Transkriptase(RT)-qPCR(quantitative Polymerasekettenreaktion)-Testung von Verstorbenen in zentralen Leichenhallen in Hamburg (Institut für Rechtsmedizin, Krematorium) auf eine SARS-CoV‑2-Infektion mittels Nasen-Rachen-Abstrich über 8 Wochen ab Auftreten pandemiebezogener Todesfälle mit vergleichender Analyse der Hell- und Dunkelfeldkollektive.

**Ergebnisse:**

Unter insgesamt 1231 verdachtsunabhängig getesteten Verstorbenen lag bei 29 Fällen (2,4 %) eine zuvor nicht bekannte SARS-CoV‑2-Infektion vor. In der ersten Phase der Pandemie überwogen Zufallsentdeckungen unter unklaren und nichtnatürlichen Todesfällen in der Rechtsmedizin, die vermehrt jüngere Altersgruppen, v. a. aus häuslicher Umgebung, umfassten. Im Krematorium zeigten sich mit weiterem Verlauf zunehmend Zufallsentdeckungen bei Todesfällen aus stationären Pflegeinstitutionen. Das Gesamtkollektiv wies soziodemografisch keine signifikanten Unterschiede zu einem Vergleichskollektiv bekannter/registrierter SARS-CoV‑2-assoziierter Todesfälle auf. In der Dunkelfeldkohorte war die Todesursache COVID-19 signifikant seltener.

**Schlussfolgerung:**

Ein systematisches verdachtsunabhängiges PCR-basiertes Monitoring von Todesfällen ermöglicht eine vollständigere Erfassung von SARS-CoV‑2-positiven Sterbefällen insbesondere im nichtklinischen Sektor. Durch die Erfassung eines Dunkelfelds, das einer Routineleichenschau bislang entgeht, kann ein präventiver epidemiologischer Beitrag geleistet werden.

## Abstract (en)

**Background:**

In the context of the COVID-19-pandemic, mortality and incidence are key determinants to assess the transmission dynamics and the resulting potential threat. Systematic microbiological monitoring of deaths provides a fundamental basis to particularly assess underrecording of community-acquired mortality. It should be further elucidated whether a death cohort of previously unreported cases may be structurally different from the cohort of officially registered cases.

**Methods:**

A systematic reverse transcription (RT) qPCR testing for SARS-CoV‑2 infections from nasopharyngeal swab samples was carried out. A representative sample of corpses from crematoria and the Institute of Legal Medicine of the Federal State of Hamburg were included. A comparative analysis of primarily reported and unreported fatalities in an 8‑week period after occurrence of the first pandemic-related deaths in Hamburg was performed.

**Results:**

A total of 1231 deaths were included, all of which were previously unsuspicious for SARS-CoV‑2 infection. Thereof 29 cases of previously unknown infections were recorded. In the first phase of the pandemic, incidental findings predominantly occurred among younger people from domestic environments with unclear or unnatural manner of death at the Institute of Legal Medicine. Over time, incidental findings investigated at the crematoria increased, mostly related to nursing home residents. The overall cohort showed no significant sociodemographic differences to a comparative collective of known SARS-CoV‑2-associated deaths. Primarily unreported cases showed a significantly lower proportion of COVID-19 as the underlying cause of death.

**Conclusion:**

A systematic PCR-based monitoring of deaths allows a more targeted detection and classification of SARS-CoV‑2 positive cases. A preventive contribution can be made by disclosing unreported pandemic-related cases of death.

## Einleitung

Epidemien, die die Menschheit heimsuchten und zahlreiche Todesfälle forderten, finden sich seit der Antike dokumentiert [11]. Teilweise breiteten sich diese auch als Pandemien aus. Neben Pest- und Cholerabakterien sind als Erreger der jüngeren Zeit HIV- und Influenzaviren zu nennen [20]. Seit Dezember 2019 fielen in der zentralchinesischen Provinz Hubei Pneumonien unklarer Ätiologie auf; als Erreger konnte ein neues Coronavirus (SARS-CoV‑2) identifiziert werden [7]. Ende Februar 2020 erreichte die Infektionswelle die Freie und Hansestadt Hamburg. Bis zum 20.05.2020 hatten sich 5042 Menschen infiziert und 236 Personen waren verstorben [5]. Die 7‑Tages-Inzidenz lag in Hamburg in der 13. Kalenderwoche (KW) 2020 mit ca. 50 Fällen/100.000 Einwohner deutlich über dem Bundesdurchschnitt, fiel in den folgenden 8 Wochen jedoch erheblich ab, sodass sie in der 17. KW bereits gering, in der 21. KW dann mit ca. 36 Fällen/100.000 Einwohner erheblich unterhalb des Bundesdurchschnitts lag ([21], eigene Auswertung). Die ersten beiden „Coronavirus-disease-2019“(COVID-19)-Todesfälle in Deutschland traten am 09.03.2020 in Nordrhein-Westfalen (Essen, Gangelt im Kreis Heinsberg) auf; bereits am Tag zuvor verstarb eine in Hamburg gemeldete Person während eines Ägyptenaufenthaltes an den Folgen der SARS-CoV‑2-Infektion [10]. Seit Ausrufung der Pandemie durch die Weltgesundheitsorganisation (WHO) bestehen bezüglich der zugrunde liegenden Letalität Unsicherheiten bei hoher Inkongruenz international verfügbarer Angaben [1, 12, 18, 23]. Begründet ist dies v. a. durch die nicht sicher bestimmbare Dunkelziffer der Infizierten sowie der tatsächlich der Infektion zuzuordnenden Todesfälle.

Die vorliegende Arbeit untersucht das bislang unbekannte Dunkelfeld von SARS-CoV‑2-positiven bzw. COVID-19-Todesfällen in einer großstädtischen Sterbekohorte und vergleicht dieses mit ante mortem diagnostizierten Sterbefällen infolge des Virus.

Andere Fragen stellen sich in diesem Zusammenhang bezüglich des Arbeitsschutzes für alle Berufe, die Kontakt mit Verstorbenen haben, u. a. in der Bestattungsbranche, aber auch für trauernde Angehörige eines Sterbefalls. Bei anzunehmender Kontagiosität von COVID-19-Verstorbenen könnte insbesondere von unentdeckt Infizierten eine besonders hohe Ansteckungsgefahr ausgehen [15, 16].

## Methode

Nach Bekanntwerden der ersten COVID-19-Todesfälle in Deutschland wurden im Institut für Rechtsmedizin, das als öffentliche Leichenhalle für unklare und nichtnatürliche Todesfälle in Hamburg fungiert, ab dem 24.03.2020 für ca. 8 Wochen, bis zum 20.05.2020, systematisch Nasen- und Rachenabstriche (ESwab, Fa. Copan Italia, Brescia, Italien) von eingegangenen Verstorbenen mittels Reverse-Transkriptase(RT)-qPCR(quantitative Polymerasekettenreaktion)-Testung auf SARS-CoV‑2 untersucht (Stichprobe 1). Der zeitliche Abstand zwischen Todesfeststellung und Leicheneingang im Institut wurde als postmortales Intervall dokumentiert, da der verdachtsunabhängige Abstrich zu diesem Zeitpunkt vom Team des Instituts für Rechtsmedizin angefertigt worden war. Darüber hinaus wurden zeitparallel, anlässlich der amtsärztlichen Leichenschau vor Feuerbestattung, im Rahmen einer Querschnittsstudie im Krematorium Hamburg-Öjendorf auf identische Weise alle Verstorbenen mit Hamburger Meldeadresse auf SARS-CoV‑2 gescreent (Stichprobe 2). Die Stichprobe 1 erfasste nur wenige Sterbezeitpunkte kurz vor Studienbeginn am 24.03.2020; in Stichprobe 2 gingen aufgrund der möglichen Verzögerung bis zur Krematoriumsleichenschau 29 Todesfälle ein, die sich bereits seit 01.03.2020 ereignet hatten. Ausschlusskriterien für die Testung waren ein bereits vorab bekannter positiver PCR-Status für SARS-CoV‑2 oder eine deutlich fortgeschrittene Leichenfäulnis (definiert als Durchschlagen des Venennetzes oder flächenhafte Grünfäulnis). Die Labordiagnostik fand im Institut für Medizinische Mikrobiologie, Virologie und Hygiene des Universitätsklinikums Hamburg Eppendorf (UKE) statt. In bestätigten Fällen erfolgte eine Fallmeldung an die Behörde für Gesundheit und Verbraucherschutz (BGV). Das zuständige Gesundheitsamt ordnete im Regelfall nachfolgend gemäß §25 (4) des Infektionsschutzgesetzes (IfSG) eine Obduktion an, die in der Rechtsmedizin durch eine vorausgehende Computertomographie ergänzt wurde. Auf Basis von Informationen durch Angehörige und Hausärzte und/oder Pflegedienste erfolgte eine Klassifikation nach sozioökonomischen, demografischen und medizinischen Kriterien. Die autoptisch erhobenen Befunde erlaubten die Differenzierung zwischen Todesfällen durch COVID-19 und solchen mit nachgewiesener SARS-CoV‑2-Infektion, jedoch alternativer Todesursache [6].

Statistische Vergleiche erfolgten mit 212 Sterbefällen, die im Rahmen eines Monitoring-Auftrags der Hamburger Behörde für Gesundheit und Verbraucherschutz überwiegend gezielt in das Institut für Rechtsmedizin zur weiteren Diagnostik verbracht worden waren (Stichprobe 3) – bei diesen war im Untersuchungszeitraum eine Infektion mit SARS-CoV‑2 bereits zum Todeszeitpunkt bekannt (Hellfeld).

Die Fallcharakteristika wurden deskriptiv ausgewertet (IBM SPSS Statistics v. 26). Quantitative Daten (Alter, BMI) wurden deskriptiv nach Test auf Normalverteilung mittels ANOVA für unabhängige Stichproben auf signifikante Unterschiede getestet. Kategoriale Daten (Sterbeorte) wurden je nach Geeignetheit mit Fisher’s Exact Test bzw. Chi-Square Test auf Signifikanz untersucht. Das Signifikanzniveau wurde für alle statistischen Tests mit α = 0,05 festgelegt.

## Ergebnisse

Es wurden in die Stichprobe 1231 Verstorbene eingeschlossen. Dabei handelte es sich in 59,6 % um Sterbefälle aus dem Institut für Rechtsmedizin Hamburg (*n* = 734) und in 40,4 % der Fälle um Verstorbene im Krematorium Hamburg-Öjendorf (*n* = 497).

Im Rahmen des systematischen, *verdachtsunabhängigen *Abstrichscreenings in den beiden Stichproben wurden 29 Infektionen erst postmortal detektiert (Dunkelfeld). Trotz der deutlich geringeren Fallzahl in Stichprobe 2 (Krematorium) waren hier 19 (3,8 %) dieser Fälle zuzuordnen, 10 bis dato unbekannte Infektionen (1,4 %) fanden sich in Stichprobe 1 (Rechtsmedizin). Es handelte sich in allen 10 Fällen um Einlieferungen aufgrund ungeklärter Todesart – sämtliche Todesfälle waren zu Hause tot aufgefunden worden. Staatsanwaltliche Ermittlungen wurden in 2 Fällen weitergeführt, mit Anordnung gerichtlicher Obduktionen (fraglicher Pflegefehler sowie Vorwürfe gegen Rettungswagenbesatzung bei vorausgehendem Einsatz zu Lebzeiten).

Die beiden Stichproben zeigten eine divergierende Alters- und Geschlechtsverteilung. Stichprobe 1 wies eine ausgeglichene Geschlechtsverteilung auf, während in der durch ein mehr als ein Jahrzehnt höheres Durchschnittsalter charakterisierten Stichprobe 2 Frauen erheblich überrepräsentiert waren (Tab. 1). Unter den Zufallsentdeckungen fanden sich 18 Frauen (62,1 %; F/M in Stichprobe 1: 4/6; Stichprobe 2: 14/5). Innerhalb der Stichproben unterschieden sich Zufallsentdeckungen und PCR-negative Sterbefälle von der Alters- und Geschlechterstruktur her statistisch nicht (*p* > 0,05). Gegenüber der Hellfeldkontrollgruppe gab es dagegen signifikante Unterschiede bei den durchschnittlich 10 Jahre jüngeren Zufallsentdeckungen nur in Stichprobe 1 (*p* < 0,05). Der durchschnittliche Body-Mass-Index (BMI) unterschied sich in Stichprobe 1 nicht signifikant zwischen Zufallsentdeckungen und PCR-Negativen einerseits sowie gegenüber dem Hellfeld andererseits. (In Stichprobe 2 lagen mangels Messungen von Körpergröße und Körpergewicht im Krematorium keine Daten vor.)

**Table 1:** 

	Stichprobe 1		Stichprobe 2		SARS-CoV‑2-Hellfeld
	Zufallsentd	PCR-neg	Zufallsentd	PCR-neg	
*n*	10	724	19	478	212
Geschlecht (m/w, %)	60/40	61,6/38,4	26,3/73,7	47,5/52,5	53,2/46,8
Alter (a) (Mw, StA)	67,8 (20,8)	67,9 (20,2)	84,5 (7,7)	80,2 (11,9)	79,7 (11,0)
BMI (kg/m^2^) (Mw, StA)	27,1 (9,3)	26,1 (7,9)	Unbekannt	Unbekannt	27,1 (8,5)

*BMI* Body-Mass-Index, *PCR* Polymerasekettenreaktion, *SARS-CoV‑2* „severe acute respiratory syndrome coronavirus type 2“, *m* männlich, *w* weiblich, *Mw* Mittelwert, *StA* Standardabweichung

**Figure 1:**
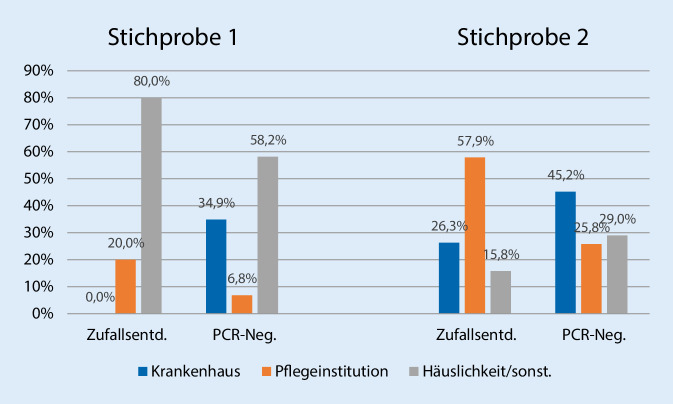

Die Zufallsentdeckungen in Stichprobe 1 waren weit überwiegend (8 von 10 Fällen = 80 %) in der eigenen Häuslichkeit verstorben, unter den PCR-Negativen dagegen nur etwa die Hälfte (53 %). In Stichprobe 2 waren die Personen mehrheitlich mit 11 von 19 Fällen (ca. 58 %) im Pflegeheim verstorben (PCR-Negative 26 %, *p* < 0,01). Insgesamt gab es nur 5 krankenhausbezogene Zufallsentdeckungen in beiden Stichproben (Abb. 1). In der Hellfeldvergleichsgruppe lag der Sterbeort mit 76,4 % weit überwiegend im Krankenhaus (*n* = 162), nur 18,9 % verstarben in Pflegeinstitutionen (*n* = 40) und lediglich 3,8 % im häuslichen Umfeld (*n* = 10).

Im Zufallskollektiv betrug das durchschnittliche postmortale Intervall 163 h (SD +/– 160 h, Spannweite 3–528 h), im Hellfeld 51 h (SD +/– 76 h, Spannweite 1–633 h).

**Figure 2:**
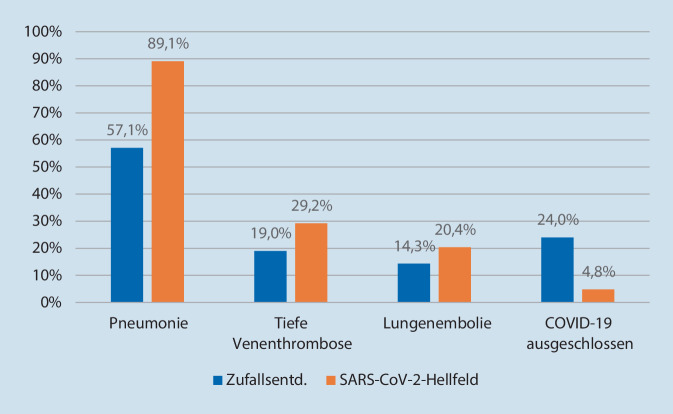

Autoptisch ließ sich in 57 % der Zufallsentdeckungen (*n* = 12 von 21 mit Obduktion, 8 Fälle blieben ohne Leichenöffnung, da Angehörigenzustimmung fehlte) ein unmittelbar COVID-19-bedingter Tod feststellen; in weiteren 19 % war ein Zusammenhang nicht auszuschließen. In 24 % dagegen bestand keine Assoziation unter aktuellem pathophysiologischen Verständnis (überwiegend kardiale Todesfälle ohne Lungenaffektion). Unter den primär COVID-19-bedingten Fällen wiesen fast alle, bis auf einen Fall, einen akuten Atemwegsinfekt auf. In 4 dieser Fälle fanden sich frische Thrombosen im venösen Gefäßsystem, die in 3 Fällen von Lungenarterienthromboembolien begleitet wurden. In der bekannt SARS-CoV‑2-infizierten Kohorte (*n* = 146 mit Obduktion) zeigten nur 4,8 % keine COVID-19-Assoziation bei der Todesursache. Es bestand bei 89,1 % eine akute Atemwegsinfektion, die mit einem todesursächlichen COVID-19-Verlauf assoziiert war; knapp ein Drittel der Fälle (29,2 %) wies tiefe Venenthrombosen und 20,4 % zudem Lungenarterienthromboembolien auf (Abb. 2).

**Figure 3:**
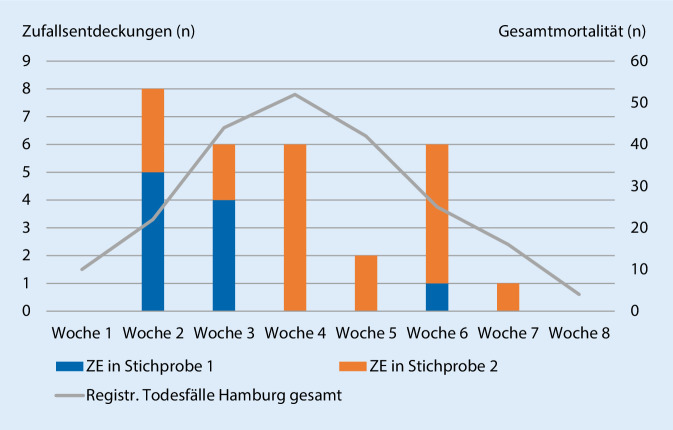

Bei Betrachtung des Zeitstrahls der Zufallsentdeckungen im Studienzeitraum fällt auf, dass diese initial v. a. in Stichprobe 1 auftraten (entsprechend der zunächst in jüngeren Altersgruppen prävalenten Infektion), ab der vierten Woche im Rahmen der Untersuchung dann – bei insgesamt sinkender Inzidenz – fast ausschließlich nur noch in Stichprobe 2 (entsprechend höherer Altersgruppen), insbesondere im Zuge der Ausbreitung der Infektion in die stationäre Pflege (Abb. 3).

Eine orientierende Hochrechnung auf die Anzahl möglicher Dunkelfeldtodesfälle mit SARS-CoV‑2-Infektion bzw. COVID-19 präsentiert sich chronologisch wie folgt (Tab. 2): Ausgehend von einer durchschnittlich zu erwartenden Sterbefallhäufigkeit in einem vergleichbaren Zeitraum zeigt sich, dass etwa 44 % der Hamburger Todesfälle durch die Zuordnungen in die 2 untersuchten Stichproben in die Studie einbezogen werden konnten; offiziell registrierte SARS-CoV‑2-positive Todesfälle wurden nicht eingerechnet. Gesetzt, dass die untersuchten Stichproben repräsentativ für das Todesfallgeschehen in Hamburg insgesamt wären (und dass die offiziellen COVID-19-Sterbefälle weitgehend als Exzessmortalität definiert wären), würden sich in linearer Hochrechnung weitere ca. 37 nichterkannte/registrierte SARS-CoV‑2-positive Todesfälle bzw. ca. 21 COVID-19-Todesfälle zusätzlich ergeben. Insgesamt lässt sich so – die in der Studie bereits erkannten Fälle einschließend – grob eine Größenordnung von 38 nicht durch die üblichen medizinischen Abläufe (ggf. Symptome → Entscheid für/gegen Arztkontakt → Exitus → Todesfeststellung →Leichenschau) entdeckten COVID-19-Todesfällen für den Untersuchungszeitraum abschätzen. Dies entspricht einer Erhöhung von ca. 16 % gegenüber dem Stand tatsächlich registrierter Todesfälle bis zum Zeitpunkt des Studienendes am 20.05.2020.

**Table 2:** 

Berechnungsgrundlage	*n*
Sterbefälle in Hamburg/Jahr (4-Jahres-Mittel 2016-2019)	17.636
Davon: Erwartungswert im Untersuchungszeitraum (58 Tage)	2802
Saisonale Anpassung durchschnittlicher Sterblichkeit, Zeitraum 24.03.2020–20.05.2020 (4-Jahres-Mittel, s. oben): minus 0,5 %	2788
Abzug, untersuchte Stichprobe, *n* = 1231; d. h. restliche, nichtuntersuchte Todesfälle	1557
Von Zufallsentdeckungsrate in hier untersuchter Stichprobe ausgehend (2,4 %): Hochrechnung auf Dunkelfeld-SARS-CoV‑2-Prävalenz bei nicht in dieser Studie untersuchten Hamburger Todesfällen in gleicher Periode	36,7
Von COVID-19-Bestätigungsrate (57,1 %) in Stichprobe ausgehende Korrektur: Hochrechnung auf weitere tatsächlich COVID-19-assoziierte Fälle bei nicht in dieser Studie untersuchten Hamburger Todesfällen in gleicher Periode	21,0
Lineare HR, gesamtes Dunkelfeld, COVID-19-Sterbefälle (57 % der entdeckten 29 SARS-CoV‑2-Fälle in Studie plus hier errechnete, geschätzte ca. 21 Fälle in Hamburg im Zeitraum 24.03.2020–20.05.2020)	Ca. 38
Abgeleitete Dunkelfeldkorrektur für Gesamtprävalenz von COVID-19-Sterbefällen (registriert in Hamburg bis 20.05.2020: n = 236 Todesfälle)	+ ca. 16 %

*SARS-CoV‑2* „severe acute respiratory syndrome coronavirus type 2“

## Diskussion

Ein gezieltes Infektion-Monitoring und ein entsprechendes, daraus abgeleitetes Containment-Management sind im Rahmen einer Pandemie von elementarer Bedeutung. Durch eine systematische Testung Verstorbener anlässlich einer zentralisierten Leichenschau ist es in zentralen Leichenhallen wie Krematorien oder in einem Organisationsmodell wie in Hamburg, mit Zentralisierung aller unklaren und nichtnatürlichen Todesfälle an einem Ort, möglich, einen relevanten Beitrag zur Erfassung der Gesamtmortalität im Rahmen der COVID-19-Pandemie oder zukünftiger Szenarien zu leisten. Entscheidend ist, dass eine ausreichende Validität von Testverfahren auch für die postmortale Anwendung bis hin zu höheren postmortalen Intervallen nachgewiesen ist [9] und eine ausreichend große, annähernd repräsentative Stichprobe erfasst werden kann. Eine Modellrechnung zeigt, dass die Studie ca. 44 % der im Untersuchungszeitraum geschätzt 2788 in Hamburg Verstorbenen (Schätzung ohne Effekt einer Übersterblichkeit durch COVID-19) inkludiert haben dürfte. Obgleich Nasen-Rachen-Abstriche auch bei Leichenschauen am Ereignisort – also außerhalb von zentralen Leichenhallen – prinzipiell durchgeführt werden könnten, würde eine ähnlich repräsentative Kohorte in der Realität sicherlich nicht zustande kommen können.

Unter den 1231 verdachtsunabhängig auf eine SARS-CoV‑2-Infektion untersuchten Verstorbenen fanden sich 2,4 % positive Fälle, die zuvor nicht als Infizierte bekannt waren. In linearer Hochrechnung kann darauf geschlossen werden, dass die Zahl der COVID-19-bedingten Todesfälle um bis zu ca. 16 % höher liegen könnte als offiziell für Hamburg bis Ende Mai 2020 registriert [5]. Auch andere Autoren thematisieren eine Mortalitätsunterschätzung, allerdings ohne dabei ein ggf. ursächliches Dunkelfeld zu berücksichtigen [12, 19]. Die vorliegende Studie bezieht sich auf 2 sehr verschieden strukturierte Kollektive, die nachweislich auch zu verschiedenen Zeitpunkten von der Infektionswelle erfasst wurden, wodurch eine unkritische Standardisierung der Hochrechnung hin zu einer „Mortalitätskorrektur“ für Hamburg und auch darüber hinaus problematisch wäre und die dezidierte Einzelfalluntersuchung von Sterbefällen durch postmortale Untersuchungen, insbesondere Obduktionen, nicht ersetzt werden kann.

Die Verteilung der Zufallsentdeckungen mit einer höheren Erfassungsquote im Krematorium lässt sich plausibel dadurch begründen, dass es sich bei den Sterbefällen, die direkt im Institut getestet wurden, um eine bereits im Vorfeld selektierte Kohorte mit einem annehmbar geringeren infektiologischem Risikoprofil handeln kann. Dennoch zeigt diese Kohorte v. a. in den ersten Wochen der epidemischen Ausbreitung – mit Betroffenheit v. a. der jüngeren Altersgruppen – eine besondere Bedeutung für die Erfassung des Gesamtbildes. Im Institut für Rechtsmedizin werden hauptsächlich ungeklärte und nichtnatürliche Todesfälle aufgenommen; einzig die im UKE Verstorbenen gehen regelmäßig auch als natürlich registrierte Sterbefälle im Institut ein. Im Krematorium Hamburg-Öjendorf werden hingegen annähernd nur Verstorbene mit bescheinigter natürlicher Todesart gesehen – diese weisen naturgemäß ein höheres Lebensalter auf und sind aufgrund damit einhergehender Komorbiditäten als typische Risikogruppe zu definieren [13].

Abzugrenzen sind für Korrekturschätzungen der Mortalität Todesfälle, bei denen SARS-CoV‑2 zum Todeszeitpunkt keine COVID-19-Erkrankung verursacht hatte, sondern nur als Zufallsbefund gelten kann. Etwa 43 % der Todesfälle der Zufallsentdeckungen waren nicht unmittelbar durch COVID-19 bedingt, im Vergleich dazu < 5 % der Hellfelduntersuchungen in der Vergleichsstichprobe. In knapp der Hälfte dieser Fälle dürfte es somit zwar zu einer SARS-CoV‑2-Infektion, jedoch nicht zur Etablierung einer letalen COVID-19-Erkrankung gekommen sein. Eine damit einhergehende, anzunehmende Symptomlosigkeit kann insofern durchaus mit der Dunkelfeldzugehörigkeit korrelieren.

Auffällig, jedoch zu erwarten, war der in der Kohorte der Zufallsentdeckungen im institutionellen Pflegesektor und in der Häuslichkeit gelegene Sterbeortschwerpunkt. Sterbefälle aus Krankenhäusern fanden sich hier erwartungsgemäß unterrepräsentiert. Bei den im Krankenhaus-Setting eingetretenen Sterbefällen, welche sich sämtlich nicht in intensivmedizinischer Behandlung befanden, handelte es sich um Patienten, die teils symptomatisch waren, jedoch in den Wochen vor dem Tod negativ via Abstrich getestet wurden, sowie um symptomfreie Fälle ohne Testhistorie. Auf die Problematik der falsch-negativen Testergebnisse von Nasen-Rachen-Abstrichen wurde bereits hingewiesen [14]; diese zeigen sowohl in frühen als auch im späten Stadium der Infektion eine erhöhte Wahrscheinlichkeit eines falsch-negativen Befundes. Postmortale Diagnostik kann theoretisch durch autolysebedingte Exposition von Viruspartikeln demgegenüber zu einem erleichterten Nachweis führen.

In einer fehlenden Testmotivation ist möglicherweise ein Erklärungsansatz für nichterkannt Infizierte aus dem häuslichen Milieu zu sehen – diese waren überwiegend hochbetagt und dürften Corona- und Lockdown-bedingt ggf. isoliert sowie nicht in adäquater medizinischer Betreuung gewesen sein. Möglicherweise handelte es sich lediglich um subklinische bzw. inapparente Krankheitsverläufe, bei denen keine Testung in Erwägung gezogen wurde und damit eine Infektion zunächst und während Lebzeiten unerkannt blieb. Mit der Einführung von Reihentests in Krankenhäusern und Pflegeeinrichtungen, unterstützt durch gesetzgeberische Maßnahmen [4], war im hier vorgestellten Gesamtkollektiv im Verlauf die Zahl der Zufallsentdeckungen rückläufig.

Für zuliefernde Institutionen, wie Polizei, Feuerwehr und medizinische Grundversorger, war die Information zum Infektionsstatus eines Verstorbenen von großem Interesse, da die tatsächlich praktizierten Schutzmaßnahmen nicht in allen Fällen dem bei SARS-CoV‑2-Infekt notwendigen Standard entsprechen konnten. Insbesondere in Situationen mit erschwertem Risikoassessment (z. B. fehlende Krankenunterlagen) und fehlender potenziell todeskausaler Risikokonstellation ist ein RT-qPCR-basiertes Screening der Todesfälle auf den neuen Erreger anzuraten. Ein klassisches Beispiel hierfür stellen plötzliche Sterbefälle im häuslichen Umfeld dar. Auch im Institut für Rechtsmedizin wurden vor dem Hintergrund des Infektions- bzw. Arbeitsschutzes RT-qPCR-Screening-Ergebnisse in die tägliche Routine miteinbezogen. So war die Durchführung einer inneren Leichenschau vom Vorliegen entsprechender Testergebnisse zum Sektionszeitpunkt abhängig und wurde bei Eilsektionen um die Durchführung antigenbasierter Schnelltests ergänzt (dies entspricht den zuletzt durch das Paul-Ehrlich-Institut publizierten Maßgaben zur Anwendung von „Point-of-care-Tests“ [17]). Bei Vorliegen eines positiven Testbefundes (RT-qPCR-/Antigentest) wurden die bei Transport und Umgang mit der Leiche angewendeten, den RKI-Empfehlungen [22] folgenden Hygienemaßnahmen für den Fall der Obduktion in Orientierung an publizierte Empfehlungen ergänzt [8]. Dies bezieht sich auf die persönliche Schutzausrüstung (Schutzbrillen, partikelfiltrierende Halbmasken der Schutzklasse FFP2, Einmalkittel, Einmalhandschuhe, OP-Haube), die Nutzung eines „Biosafety-level“(BSL)-2-konformen Sektionssaals (10-facher Luftaustausch, „Untertischabsaugung“), die Desinfektion von Flächen sowie Arbeitsgeräten gemäß angepasstem Hygieneplan, die Entsorgung von festen Abfällen in vorgesehenen Tonnen zur Inaktivierung mit geeigneten Maßnahmen und den betriebsinternen Transport von nichtinaktivierten infektiösen Materialien/Asservaten in sicheren Gefäßen [2, 3].

Mit den hier vorliegenden Ergebnissen ist, insbesondere in pandemischen Zeiten, für den Bereich der Leichenschau und der Logistik im Umgang mit Verstorbenen außerhalb des Sektionssaals an eine erhöhte Sorgfalt und an hohe Ansprüche an den Eigen- und Arbeitsschutz für Mitarbeiter beim Umgang mit Leichen zu appellieren – die aktuellen Empfehlungen des Robert Koch-Institutes berücksichtigend [18].

Trotz drastischer Maßnahmen seitens der Politik sowie intensivster behördlicher und medizinisch-interdisziplinärer Bemühungen lassen sich Infektionsausbrüche nicht vollständig überwachen und der Infektionsverlauf nur vage vorhersagen. Die Notwendigkeit des Monitorings des Mortalitätsverlaufs sowie der Minimierung eines Dunkelfelds sollte im Interesse der Gesundheitsbehörden und auch der Angehörigen sein. Im Rahmen einer nicht kontrolliert erscheinenden Infektionsdynamik können Reihentestungen v. a. bei Verstorbenen im nichtklinischen Sektor einen wichtigen Beitrag zur gezielten Überwachung eines Pandemiegeschehens darstellen – entweder im Zuge weiterer SARS-CoV‑2-Infektionswellen oder aber im Falle einer anders gelagerten zukünftigen Pandemie.

## Fazit für die Praxis

- Ein SARS-CoV‑2-Screening in Leichenschaukollektiven ist sinnvoll und organisatorisch in großen Leichenhallen/Krematorien gut umsetzbar.
- Reihentestungen stellen, v. a. bei Verstorbenen im nichtklinischen Sektor, einen wichtigen Beitrag zu gezielter Überwachung sowie Eingrenzung eines Pandemiegeschehens dar.
- Je nach Infektiosität eines Erregers ist ein solches Screening auch arbeitsschutzrelevant.
- In einer deutschen Metropole wie Hamburg, die in den ersten Wochen der Studie zu den Regionen mit hoher Inzidenz gehörte, ist im Zeitraum eines dynamischen Infektionsgeschehens von einer Untererfassung pandemiebezogener Todesfälle in der Registrierung der öffentlichen Gesundheitsdienste auszugehen.
